# Measuring body core temperature using a novel non-invasive sensor

**DOI:** 10.1186/2046-7648-4-S1-A28

**Published:** 2015-09-14

**Authors:** Yoram Epstein, Savyon Mazgaoker, Doron Gruber, Daniel S Moran, Ran Yanovich, Itay Ketko, Yuval Heled

**Affiliations:** 1 Heller Institute of Medical Research, Sheba Medical Center, Tel Hashomer, Israel; 2 Sackler Faculty of Medicine, Tel Aviv University, Tel Aviv, Isreal; 3 Ariel University and Washington College of Education, Israel

## Introduction

In various jobs workers may be exposed to extreme environmental conditions and physical activities. Under these conditions it is imperative to follow body temperature in workers in order to protect them from overheating leading to heat related injuries. The Dräger Double Sensor (DS) is a novel non-invasive device based on heat flux balance approach for the assessment of body core temperature [1]. The purpose of this study was to compare DS measurements to rectal temperatures and to evaluate the agreement between the two measurements.

## Methods

17 male subjects dressed in shorts performed the following experimental protocol: 30 min rest under thermal comfort conditions, 30 min rest under hot climate (40 °C, 40 % rh) and 60 min of exercise under the hot climatic conditions. Continuous measurements were obtained with the DS (T_DS_) in parallel to rectal temperature (T_re_) (YSI-401 thermistor).

## Results

**Figure 1:**
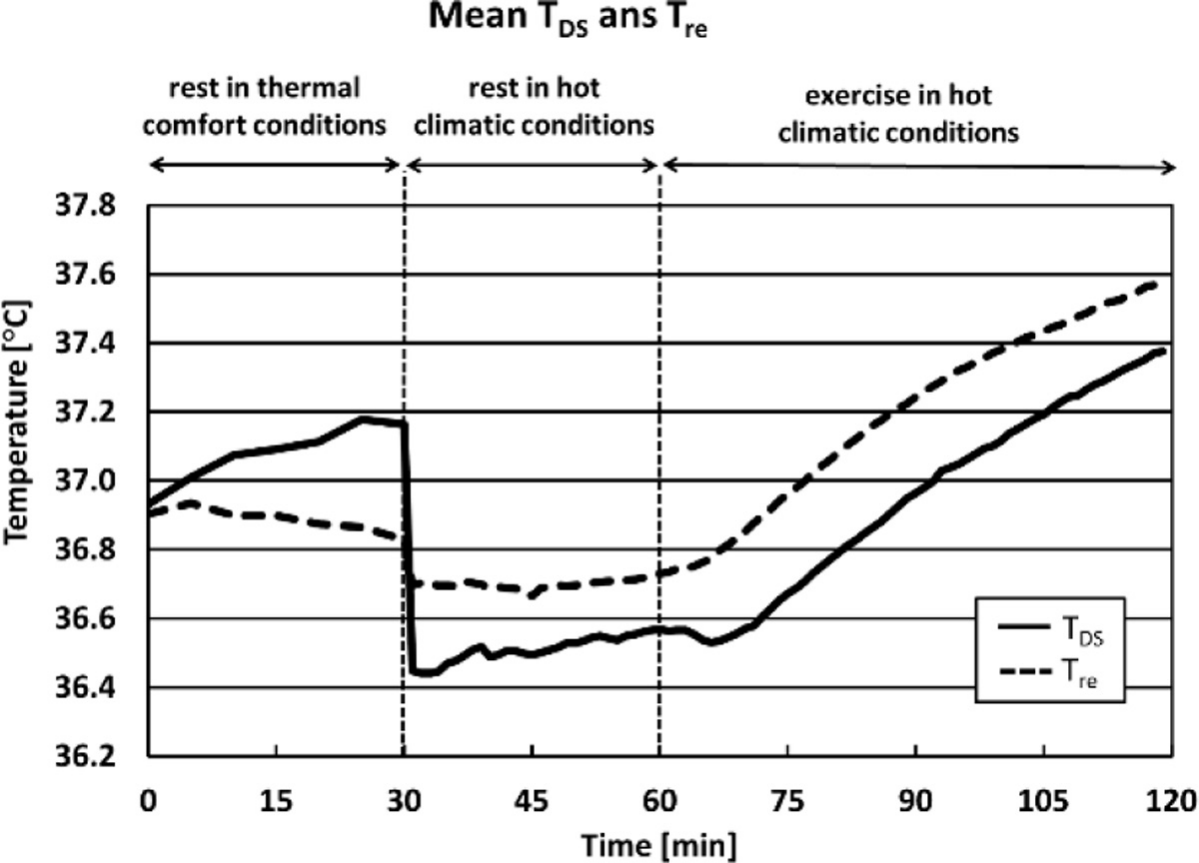


**Figure 2:**
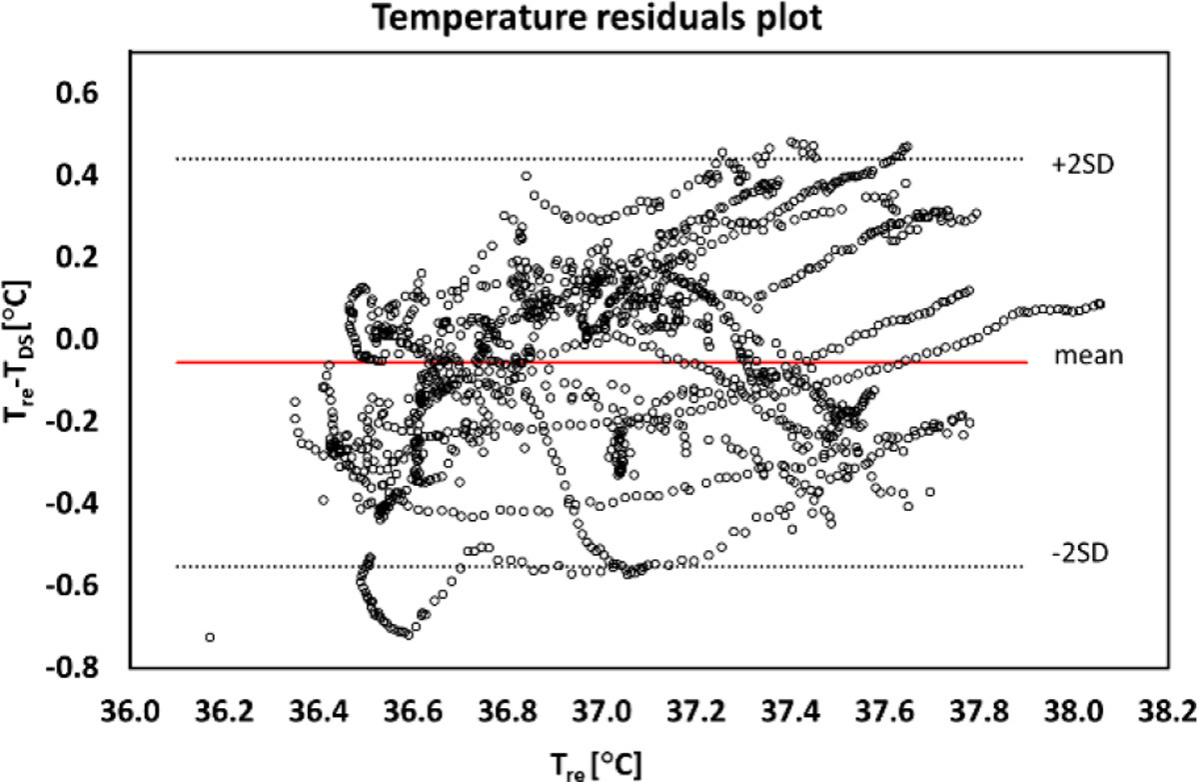


During rest under comfortable climatic conditions T_DS _tended to be lower than T_re _(Figure 1). During heat exposure, mean T_DS _was within +0.3 °C of mean T_re_. A good linear correlation (r = 0.99) between the T_DS _and T_re _during exercise in the heat was found, which enabled to adjust T_DS_. A scatter plot of Temperature residuals (T_re_-T_DS_) of the corrected data was within ±0.5 °C of mean residual (Figure 2).

## Discussion

T_DS _is within a reasonable range from the "gold standard" (T_re_) during heat stress. It seems that T_DS _equilibrates slower than T_re _and, therefore, the agreement between the two measurements is low during the first part of the exposure (30 minutes).

## Conclusion

The results are promising for potential use of the DS in workers under field conditions, especially under environmental heat stress and when dressed in protective garments. Further investigations are required to validate the data under various conditions (e.g. higher heat stress).
